# Frequency and clinical characteristics of HER2 over-expressed breast cancer in Saudi Arabia: a retrospective study

**DOI:** 10.1186/s12905-020-01159-3

**Published:** 2021-01-06

**Authors:** Jamal Zekri, Ahmed Saadeddin, Hulayel Alharbi

**Affiliations:** 1 King Faisal Specialist Hospital and Research Centre, College of Medicine, Alfaisal University, Jeddah, Saudi Arabia; 2 King Abdulaziz Medical City, Ministry of National Guard, Health Affairs, Riyadh, Saudi Arabia; 3 King Fahad Specialist Hospital, Dammam, Saudi Arabia

**Keywords:** Breast cancer, HER2, Saudi Arabia, Epidemiology

## Abstract

**Introduction:**

This study aimed to determine the frequency of human epidermal growth factor receptor 2 (HER2) over-expression in newly diagnosed breast cancer (BC) patients in Saudi Arabia and to assess the clinical characteristics and outcomes in patients with HER2-positive disease.

**Methods:**

In the first part of the study, we retrospectively reviewed the pathology records of all patients diagnosed with BC between 2007 and 2013 at 3 hospitals in the largest 3 cities in Saudi Arabia to determine the frequency of HER2 over-expression. In the second part, a representative sample from the patients identified with HER2 over-expressed BC was selected for further investigation. Data collected included demographic and clinical characteristics such as hormone-receptor status, treatment regimens, survival data, response to treatment, and selected adverse events.

**Results:**

1867 BC records were included in the study. HER2 was overexpressed in 559 patients (29.9%); of those, 348 HER2-positive BC patients were included in subsequent analyses. In the sample of HER2-positive BC patients, median age at diagnosis was 46 years, 0.9% were male, 92.5% were Saudi, 42.4% were Hormone Receptor-negative, and 13.1% had stage IV tumors. Most patients (84.2%) underwent curative intent surgery and 71.8% received radiotherapy. Average tumor size was 3.5 ± 2.5 cm and infiltrating ductal carcinoma was the most common pathology (92.9%). As for pharmacological therapy, the most commonly used regimens were Chemotherapy + Trastuzumab combination (79.1%) in neoadjuvant setting, Hormonotherapy alone (56.2%) in adjuvant setting, and Chemotherapy + Targeted therapy combination (64.8%) as palliative treatment. At the last patient evaluation, 36.9% had complete response, while 33.2% had progressive disease. Median overall survival (OS) and progression-free survival (PFS) were not reached in patients on neoadjuvant/adjuvant pharmacotherapy. As for patients on palliative intent pharmacotherapy, median OS and PFS were 64.7 and 29.3 months respectively.

**Conclusion:**

This study provided updated figures regarding HER2 overexpression in BC in Saudi Arabia: HER2 overexpression rate (29.9%) was within the range reported in previous studies. Patients’ demographic and clinical characteristics were also similar to those reported earlier, with a median age at diagnosis of 46 years and one third of patients having locally advanced/metastatic disease at diagnosis.

## Background

Breast cancer (BC) is the second most commonly diagnosed cancer in the world and the most commonly diagnosed cancer and the leading cause of cancer death among females. More than two million new cases of BC are diagnosed annually, accounting for almost 1 in 4 cancer cases among women and 15% of cancer mortality in both developed and developing countries [1].

Age at diagnosis varies substantially between industrialized and Arab countries; in USA and Europe, the median age at presentation is around 63 years [2]. However, in the Arab countries, almost 50% of the cases are diagnosed at age below 50 (median age at diagnosis is 49.8 years). As for the stage of the disease, BC is more commonly diagnosed in advanced and metastatic stages in developing than in developed countries [3].

The situation in Saudi Arabia is no exception from other parts of the Arab world, where BC accounts for 30.1% of female cancers [4]. The Saudi National Cancer Registry reported an incidence of 127.8 BC cases per 100,000 women and a mortality rate of 25.5 per 100,000 between 2000 and 2004 [5]. In addition, most patients are diagnosed under 50 years [6] and one fourth under 40 [7] and 15% present with distant or metastatic disease [4].

Many prognostic and predictive biomarkers are utilized in BC, among which the estrogen receptor (ER), progesterone receptor (PR) and human epidermal growth factor receptor 2 (HER2) have been validated and are currently used in routine clinical practice for decision-making regarding specific treatments in BC [8].

HER2 gene is a proto-oncogene encoding the HER2 receptor. HER2 gene amplification has been identified in 25 to 30% of primary BCs [9]. Patients with HER2-positive disease are more likely to develop disease recurrence and to have a shorter overall survival (OS) and an overall poor prognosis, but these patients can benefit from specific anti-HER2 targeted therapies that can improve their outcomes [8] such as trastuzumab [10], lapatinib, pertuzumab, and other novel anti-HER2 targeted therapies [11].

As per the American Society of Clinical Oncology/College of American Pathologists (ASCO/CAP) guidelines, HER2 testing is recommended in primary invasive and metastatic BCs. Tissue can be obtained through either core or excisional biopsies. Two tests are currently validated and used for HER2 status testing: immunohistochemistry (IHC) assay for HER2 protein overexpression and in situ hybridization (ISH) assay for HER2 gene amplification [12].

HER2 overexpression has been evaluated in several studies in Saudi Arabia. One study compared consecutive BC series from Switzerland and Saudi Arabia between 1988 and 2002 and reported a 31% frequency of HER2 amplification in Saudi Arabian BCs, which was significantly higher than that reported in the Swiss samples (17%) [13]. Another study reported a 28.3% rate of HER2 overexpression between 2000 and 2004 and found no correlation between HER2 status and other prognostics factors [14].

The results reported in the aforementioned studies were not always consistent. Moreover, data collected covered the period between 1988 until 2008 and most studies sampled only one center in Saudi Arabia. The number of drugs licensed for HER2 over-expressed BC is expanding across all treatment settings (neoadjuvant, adjuvant and palliative). Studying the frequency and the outcome of HER2 over-expressed BC can help health economists, funding agencies and decision makers draw better and more evidence-based strategies [15, 16].

The current study is structured in 2 parts utilizing combined data from three major centers in Saudi Arabia, to provide a greater representation of the frequency of HER2 overexpression in the Saudi Arabian BC population in part 1. In addition, data collection covered the period extending from 2007 until 2013, to provide an updated and accurate estimation of the frequency of HER2 overexpression figures. In part 2 of the study, we aim to present an in-depth description of the clinic-pathological features, management and the clinical outcome of HER2-positive BC in Saudi Arabia.

## Methods

This was a retrospective multicenter two-part study, consisting of chart review of newly diagnosed BC patients previously treated in routine clinical practice setting. The study was conducted in three pre-selected sites in Saudi Arabia: King Faisal Specialist Hospital and Research Center Gen.Org. (Jeddah), King Abdullah International Medical Research Center [KAIMRC]—King Abdulaziz Medical City (Riyadh), and King Fahad Specialist Hospital (Dammam). Data was collected between October 2016 and October 2018.

### Study part 1

The first part of the study aimed to determine the frequency of HER2 overexpression and included male and female adult patients (> 18 years) residing in Saudi Arabia, who were newly diagnosed with early or advanced BC between 2007 and 2013, and who had available HER2 testing results. The only reason for exclusion was having an inconclusive or equivocal result of HER2 overexpression.

The pathology records of all eligible patients at the 3 pre-selected sites during the pre-defined period were retrospectively reviewed for HER2 status determination. HER2 overexpression was determined based on the treating physician’s assessment of the pathology report regardless of the type of tissue sampled; HER2 testing methods included IHC and fluorescent in situ hybridization (FISH).

### Study part 2

In the second part of the study, a representative sample was selected from all patients with HER2-positive BC identified in part 1 of the study, and medical charts of this sample were retrospectively reviewed for the collection of data. The second part aimed to describe the demographic and clinical characteristics, as well as the hormone receptor status, treatment regimens, survival, response to treatment, and adverse events (AEs) in the sample of patients with HER2-positive disease only.

Data collected in the second part of the study included patient demographic and anthropometric characteristics (sex, age, nationality, body mass index [BMI]), site of tumor tissue sampling and HER2 testing method, ER and PR tests’ results, patient clinico-pathologic profile (date of diagnosis, primary cancer site, BC stage and metastatic sites, performance status), and comorbidities. The treatment settings (neoadjuvant, adjuvant, and palliative setting) were also recorded. Additionally, data on radical radiotherapy, pharmacological therapy (regimens) and surgery was collected. Response to treatment and patient outcome expressed as progression-free survival [PFS] and overall survival [OS] was calculated. We also recorded the patients’ status at cut-off point and safety data (only AEs reported in patients while receiving trastuzumab were collected).

### Study endpoints

The primary endpoint was the frequency of HER2 overexpression in patients newly diagnosed with early or advanced BC in the 3 study sites.

Secondary endpoints were studied in a sample of HER2-positive BC patients and included:

- Patients’ demographic and clinical characteristics
- The percentage of patients with ER/PR positive BC
- The type of treatment regimens used in the management of BC
- Median OS and PFS and the percentage of patients with complete response, partial response, progressive disease and stable disease
- Safety data (AEs reported in patients while receiving trastuzumab with or without other concomitant systemic therapies).

### Statistical methods

#### Sample size justification

No pre-estimated sample size was provided for part 1 of the study. The determination of the frequency of HER2 overexpression was based on the review of pathology reports of all patients newly diagnosed with BC between 2007 and 2013 and who had available HER2 testing results in the pre-selected sites. For part 2 of the study, a representative sample from all HER2-positive BC patients was selected. The selection of these cases was based on the integrity and completeness of data. Patients who had significant missing information precluding meaningful analysis were excluded. Examples of such patients include those who transferred their care to another institution shortly after diagnosis, refused standard systemic anti-cancer therapy recommendations and those with missing or unobtainable medical records for any reason.

#### Statistical analyses

All statistical analyses were performed using IBM SPSS Statistics for Windows, Version 22.0. Armonk, NY: IBM Corp.

Qualitative variables were summarized using descriptive statistics and were reported as frequencies and percentages. Quantitative variables were presented as means and standard deviations. Confidence intervals were two sided and provided at the 95% confidence level. Significance level was set at 5% and *p*-value < 0.05 was considered as significant. No missing data were imputed. For the OS and PFS analyses, patients were classified based on the first treatment they received after diagnosis: curative intent treatment (neoadjuvant or adjuvant therapy) or palliative intent treatment (treatment for metastatic disease). Survival data were estimated by Kaplan Meier approach and graphically presented. Log Rank test (Mantel-Cox) was used to compare the survival distributions in patients on curative versus palliative treatment. Patients who had not received pharmacotherapy were excluded from the OS and PFS analyses, since they could not be categorized as palliative/curative. The frequency of AEs was summarized according to the System Organ Classification (SOC) and within each SOC, by MedDRA preferred term.

## Results

### Study part 1

**Fig. 1:**
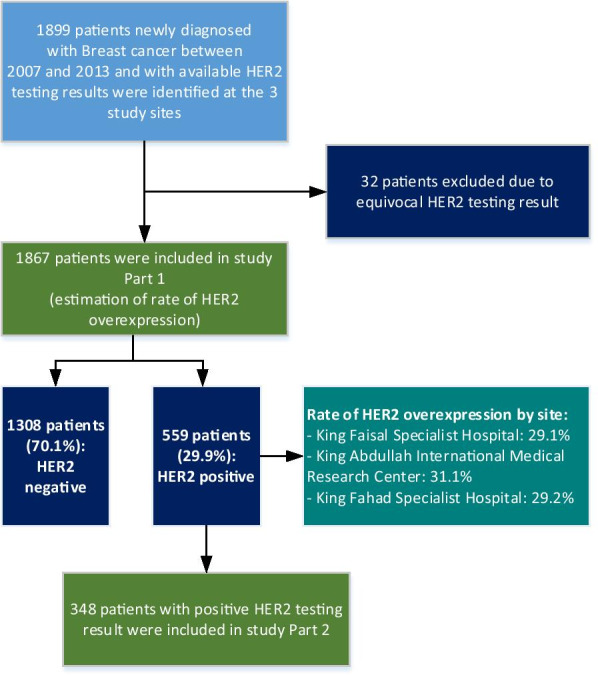
Study flowchart

From the 3 study sites, 1867 BC records with available HER2 testing results were included. HER2 was overexpressed in 559 patients (29.9%) (Table 1). Of the 559 HER2-positive BC cases identified in part 1, 348 patients were included in part 2 of the study (Fig. 1).

**Table 1:** HER2 status in patients with available HER2 testing results between 2007 and 2013

N	Positive	Negative	Total
	559	1308	1867
HER2 status with 95% CI	29.9% (27.9–32.0%)	70.1% (68.0–72.1%)	100%
*Per site distribution: n (%)*			
King Faisal Specialist Hospital and Research Center Gen. Org. (Jeddah)	210 (29.1%)	511 (70.9%)	721 (100%)
King Abdullah International Medical Research Center [KAIMRC]—King Abdulaziz Medical City (Riyadh)	237 (31.1%)	526 (68.9%)	763 (100%)
King Fahad Specialist Hospital (Dammam)	112 (29.2%)	271 (70.8%)	383 (100%)

*CI* confidence interval

### Study part 2

The median age of patients was 46 years at diagnosis and 52 years at study inclusion. Only 3 patients were male (0.9%), and the majority of patients were Saudi Arabian (92.5%). Of the total of 348 patients, 139 (39.9%) had a comorbidities, the most common of which were hypertension (22.7%), diabetes mellitus (22.1%), dyslipidemia (18.7%), hypothyroidism (9.5%), and ischemic heart disease (4.1%). In HER2-positive BC patients, 42.4% were ER and PR-negative (Table 2).

**Table 2:** Demographic and clinical characteristics of HER2 positive Breast Cancer Patients

Demographic and clinical characteristics	Total
	N = 348
*Age at study inclusion (years), n = 348*	
Mean ± SD	54.07 ± 11.55
*Age at breast cancer diagnosis (years), n = 344*	
Mean ± SD	48.14 ± 11.67
*Sex, n = 348*	
Male	3 (0.9%)
Female	345 (99.1%)
*Nationality, n = 348*	
Saudi Arabian	322 (92.5%)
Other	26 (7.5%)
*Body Mass Index (kg/m*^*2*^*), n = 262*	
Mean ± SD	30.56 ± 6.35
*Breast cancer stage based on TNM classification at diagnosis, n = 260*	
Stage I	19 (7.3%)
Stage II	103 (39.6%)
Stage III A	57 (21.9%)
Stage III B	47 (18.1%)
Stage IV	34 (13.1%)
*Metastatic sites, n = 30*	
Bone	15 (50.0%)
Lung	13 (43.3%)
Liver	13 (43.3%)
Lymph node	4 (13.3%)
Brain	3 (10.0%)
Ovaries	1 (3.3%)
Peritoneum	1 (3.3%)
*Primary cancer site, n = 348*	
Right	175 (50.3%)
Left	164 (47.1%)
Bilateral	8 (2.3%)
Unknown	1 (0.3%)
*Performance status, n = 187*	
Performance status = 0	20 (10.7%)
Performance status = 1	122 (65.2%)
Performance status = 2	31 (16.6%)
Performance status = 3	9 (4.8%)
Performance status = 4	5 (2.7%)
Performance status = 5	0 (0.0%)
*Site of biopsy, n = 348*	
Primary tumor site	328 (94.3%)
Metastatic tumor site	15 (4.3%)
Unknown, not recorded	5 (1.4%)
*HER2 test method, n = 348*	
FISH	53 (15.3%)
IHC	294 (84.7%)
*Curative intent surgery performed, n = 348*	
Yes	293 (84.2%)
No	55 (15.8%)
*If Yes, tumor size (cm), n* = *264*	
Mean ± SD	3.5 ± 2.5
*Breast cancer pathology, n* = *283*	
Infiltrating ductal carcinoma	263 (92.9%)
Infiltrating lobular carcinoma	0 (0.0%)
Medullary carcinoma	0 (0.0%)
Inflammatory breast cancer	2 (0.7%)
Tubular carcinoma	1 (0.4%)
Mucinous carcinoma	2 (0.7%)
Ductal carcinoma in situ	13 (4.6%)
Invasive ductal carcinoma	2 (0.7%)
*Hormone-receptor status, n* = *340*	
ER-/PR-	144 (42.4%)
ER + and/or PR +	196 (57.6%)
*Tumor margins, n* = *265*	
Negative	245 (92.5%)
Positive	20 (7.5%)
*More than one tumor in the breast, n = 346*	
Yes	58 (16.8%)
No	288 (83.2%)
*If Yes, tumor origin, n* = *53*	
Multifocal breast cancer	33 (62.3%)
Multicentric breast cancer	20 (37.7%)
*Lymphovascular invasion, n = 289*	
Yes	121 (41.9%)
No	168 (58.3%)
*Axillary lymph nodes examined (sentinel, sampling, dissection), n = 313*	
Yes	256 (81.8%)
No	57 (18.2%)
*Number of lymph nodes examined, n = 254*	
Mean ± SD	14.5 ± 11.2
*Number of lymph nodes involved, n = 253*	
Mean ± SD	3.7 ± 7.6
*Clinical response at last patient evaluation, n = 217*	
Stable disease	61 (28.1%)
Progressive disease	72 (33.2%)
Partial response	4 (1.8%)
Complete response	80 (36.9%)

*SD* standard deviation, *FISH* fluorescent in situ hybridization, *IHC* ImmunoHistoChemistry, *ER* estrogen receptor, *PR* progesterone receptor

Based on the TNM classification at diagnosis, 21.9% of patients had Stage III A, 18.1% had Stage III B, and 13.1% had Stage IV tumors. Data on site of biopsy and HER2 testing method, as well as primary cancer site, metastatic sites, and performance status are summarized in Table 2.

The majority of patients underwent curative intent surgery (n = 293; 84.2%) and 249 (71.8%) received postoperative adjuvant radiotherapy. Average tumor size was 3.5 ± 2.5 cm. Infiltrating ductal carcinoma was the most common form of BC (92.9%). Tumor margins were negative (clean, clear, not involved) in 92.5% of the cases. Details on tumor characteristics, lymph node status, and clinical response at last patient evaluation are present in Table 2.

As for pharmacological therapy, 110 patients received neoadjuvant pharmacotherapy, 153 received adjuvant pharmacotherapy, and 46 patients received palliative pharmacotherapy as their first treatment, while 39 patients did not receive pharmacotherapy during follow-up. Further details on pharmacotherapy in different treatment settings are present in Table 3.

**Table 3:** Pharmacological treatments and radiotherapy in HER2 positive Breast Cancer Patients

	n (%)
*Pharmacological treatment setting from diagnosis till end of data collection (cut-off point), n = 309*	
Neoadjuvant only	10 (3.2%)
Adjuvant only	134 (43.4%)
Metastatic only	46 (14.9%)
Neoadjuvant + Adjuvant + Metastatic	22 (7.1%)
Neoadjuvant + Adjuvant	74 (23.9%)
Adjuvant + Metastatic	19 (6.1%)
*Neoadjuvant treatment regimens, n = 110*	
Neoadjuvant + Metastatic	4 (1.3%)
Hormonotherapy alone	3 (2.7%)
Chemotherapy alone	83 (75.5%)
Trastuzumab alone	7 (6.4%)
Chemotherapy + Trastuzumab	87 (79.1%)
Other regimens	2 (1.8%)
*Adjuvant treatment regimens, n = 249*	
Hormonotherapy alone	140 (56.2%)
Chemotherapy alone	113 (45.4%)
Trastuzumab alone	96 (38.6%)
Chemotherapy + Trastuzumab	129 (51.8%)
Hormonotherapy + Trastuzumab	22 (8.8%)
*Metastatic treatment regimens, n = 91*	
Hormonotherapy alone	41 (45.1%)
Chemotherapy alone	40 (44.0%)
Targeted therapy alone	36 (45.1%)
Chemotherapy + Targeted therapy	59 (64.8%)
Hormonotherapy + Targeted therapy	14 (15.4%)
Hormonotherapy + Chemotherapy + Targeted therapy	2 (3.3%)
*Radiotherapy, n = 347*	
No	98 (28.2%)
Yes	249 (71.8%)

The most commonly used regimen in neoadjuvant setting was Chemotherapy + Trastuzumab combination (79.1%), while in adjuvant setting, the most commonly used regimen was Hormonotherapy alone (56.2%). As for palliative therapy, the most commonly used regimen was Chemotherapy + Targeted therapy combination (64.8%).

At the last patient evaluation available at end of data collection, 36.9% of patients had complete response, while 33.2% had progressive disease.

**Fig. 2:**
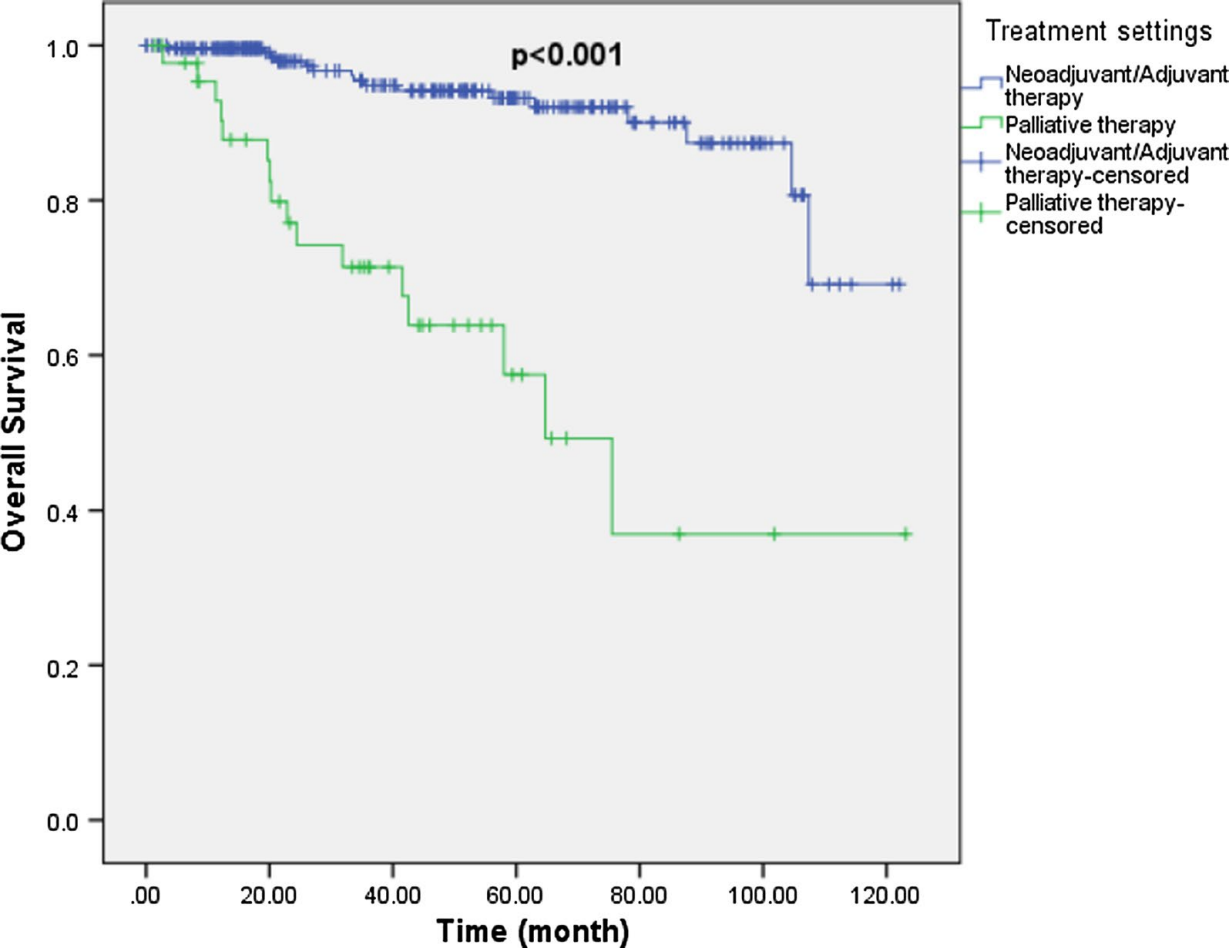
Overall survival by treatment intent

305 patients were included in the OS analysis. Of those who started on curative intent pharmacotherapy (Neoadjuvant/Adjuvant setting; n = 259) after diagnosis, 16 patients died during follow-up. Median OS was not reached in this group. As for patients who started on palliative intent pharmacotherapy (treatment of metastatic disease; n = 46) after diagnosis, 16 patients died during follow-up and median OS was 64.7 months (Fig. 2).

**Fig. 3:**
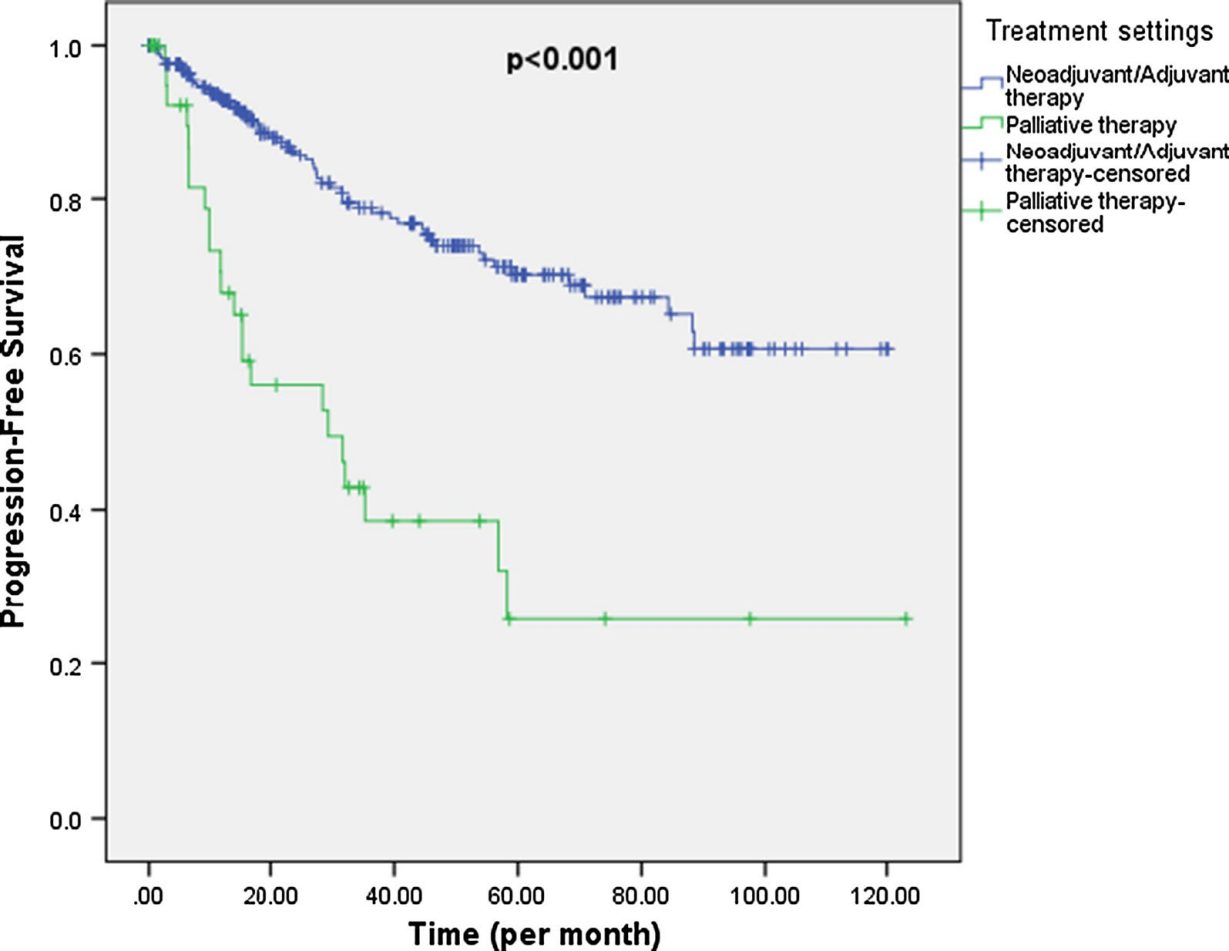
Progression-free survival by treatment intent

302 patients were included in the PFS analysis. For those who started on curative intent pharmacotherapy (Neoadjuvant/Adjuvant setting; n = 256) after diagnosis, 57 patients had documented progression during follow-up. Median PFS was not reached in this group. As for patients who started on palliative intent pharmacotherapy (treatment of metastatic disease; n = 46) after diagnosis, 23 patients had documented progression during follow-up and median PFS was 29.3 months. The difference in PFS distribution between the 2 groups was highly significant (p < 0.001) (Fig. 3).

17 patients were reported to have experienced SAEs (4.9%) and 83 patients to have experienced AEs during the administration of trastuzumab. The most commonly reported AEs were decreased ejection fraction (7.2%), stomatitis (3.7%), and vomiting (2.9%). Other AEs are detailed in Table 4.

**Table 4:** Adverse events experienced during trastuzumab treatment in patients with HER2-positive breast cancer

System organ class	n (%)
MedDRA preferred term	
*Any system organ class*	
*Total*	83 (23.9%)
*Investigations*	24 (6.9%)
Ejection fraction decreased	25 (7.2%)
Hepatic enzymes increased	1 (0.3%)
*Blood and lymphatic system disorders*	15 (4.3%)
Neutropenia	9 (2.6%)
Febrile neutropenia	8 (2.3%)
Lymphedema	1 (0.3%)
*Gastrointestinal disorders*	15 (4.3%)
Stomatitis	13 (3.7%)
Vomiting	10 (2.9%)
Abdominal pain upper	2 (0.6%)
Abdominal pain	1 (0.3%)
Dysphagia	1 (0.3%)
Gastritis	1 (0.3%)
Loose tooth	1 (0.3%)
*General disorders and administration site conditions*	14 (4.0%)
Pyrexia	9 (2.6%)
Pain	5 (1.4%)
Fatigue	1 (0.3%)
*Cardiac disorders*	16 (4.6%)
Cardiomyopathy	8 (2.3%)
Dyspnea	6 (1.7%)
Cardiac failure congestive	2 (0.6%)
Bradycardia	1 (0.3%)
Congestive cardiomyopathy	1 (0.3%)
Palpitations	1 (0.3%)
*Nervous system disorders*	9 (2.6%)
Neuropathy peripheral	6 (1.7%)
Dizziness	2 (0.6%)
Hypoesthesia	1 (0.3%)
Muscular weakness	1 (0.3%)
*Musculoskeletal and connective tissue disorders*	7 (2.0%)
Pain in extremity	5 (1.4%)
Arthralgia	2 (0.6%)
Back pain	1 (0.3%)
Bone pain	1 (0.3%)
*Respiratory, thoracic and mediastinal disorders*	6 (1.7%)
Cough	3 (0.9%)
Pleural effusion	1 (0.3%)
Pneumonitis	1 (0.3%)
Pulmonary embolism	1 (0.3%)
*Infections and infestations*	4 (1.1%)
Abscess limb	1 (0.3%)
Bronchitis	1 (0.3%)
Cellulitis	1 (0.3%)
Respiratory tract infection	1 (0.3%)
*Injury, poisoning and procedural complications*	4 (1.1%)
Cardiotoxicity	4 (1.1%)
*Skin and subcutaneous tissue disorders*	4 (1.1%)
Alopecia	1 (0.3%)
Dry skin	1 (0.3%)
Pigmentation disorder	1 (0.3%)
Pruritus	1 (0.3%)
Skin lesion	1 (0.3%)
*Immune system disorders*	3 (0.9%)
Hypersensitivity	2 (0.6%)
Skin reaction	1 (0.3%)
*Renal and urinary disorders*	3 (0.9%)
Flank pain	2 (0.6%)
Dysuria	1 (0.3%)
Urinary tract infection	1 (0.3%)
*Reproductive system and breast disorders*	2 (0.6%)
Endometrial hyperplasia	1 (0.3%)
Hematuria	1 (0.3%)
*Ear and labyrinth disorders*	1 (0.3%)
Otitis externa	1 (0.3%)
*Metabolism and nutrition disorders*	1 (0.3%)
Decreased appetite	1 (0.3%)
*Vascular disorders*	1 (0.3%)
Hot flush	1 (0.3%)

Frequency of AEs is sorted in descending order in the total column

A patient with multiple occurrences of an AE for a preferred term or system organ class is counted only once

A patient with multiple AEs within a primary system organ class is counted only once in the total row

## Discussion

Data on HER2 overexpression in Saudi Arabia reported in the literature mainly covers the period from 1988 until 2008. This study provided updated figures on the rate of HER2 overexpression in the Saudi Arabian BC population, for the period between 2007 and 2013.

HER2 overexpression rate was found to be 29.9% in our sample of newly diagnosed BC patients. The American Cancer Society has reported the HER2 overexpression rate in the USA to be 17% in 2017–2018 [17], while several studies in Middle Eastern and Arab populations suggest much higher rates in those populations. Al-Tamimi et al. reported a HER2 overexpression rate of 28% in a sample of Saudi Arabian women [18]. Alnegheimish et al. reported a HER2 overexpression rate of 26.8% in a retrospective study done in Riyadh between 2010 and 2014 [19]. Other studies such as Chahine et al. reported even higher rates (39.7%) in Lebanese patients between 2008 and 2013 [20]. One possible explanation for the higher HER2 overexpression rate in our study, beside potential ethnic variability, is the younger age at presentation for BC patients in our region. HER2 overexpression has been suggested to be associated with younger patient age in several studies [21–23].

As for age at diagnosis, the mean age of 48 years reported in our study is in line with numerous studies which reported similar values, among which is Saggu et al. [24] who reported a mean age of 49 years, using data from the Saudi Cancer Registry (1990–2010), in addition to other studies conducted in 2012–2014 and who have reported the same mean age of 48–50 years at diagnosis [7, 25]. Averages reported in the US and Europe are more than 10 years above this number [26]. In the United States, 65.1% of BC cases occur in women older than 55 years, based on SEER data for 2001–2005 [27]. Najjar et al. attributed this difference to social, economic and population differences between Arab and Western populations [28]. This younger age at diagnosis should encourage Arab countries, including Saudi Arabia, to reconsider the age range of the target population of national BC screening programs.

Regarding hormone-receptor status, our study has reported that 54% of HER2-positive patients were also ER-positive, while 42% were ER and PR-negative. Rudat et al. has reported higher rates of ER-positive tumors (67%), however, that study has examined hormone-receptor status regardless of HER2 receptor status [7]. Mohamed et al. [29] reported that 38% of patients with HER2-positive tumors were hormone-receptor negative, which is similar to the rate reported in our study.

Stage of disease at diagnosis was reported to be 39% and 13% for stages 3 and 4 in this study. Rudat et al. reported that 40% and 9% of BC patients at a tertiary care center in the Eastern province of Saudi Arabia had stage 3 and 4 tumors respectively [7], while another study reported that 30% and 16% of BC tumors in Saudi Arabia were stages 3 and 4 tumors respectively [26].

In our sample of HER2-positive BC patients, 84% and 72% of patients underwent curative intent surgery and postoperative adjuvant radiotherapy respectively. However, the type of surgery was not specified (breast-conserving or radical mastectomy) in our study, which might preclude comparison to other figures reported in similar studies.

The large tumor size at diagnosis observed in this study (3.5 cm) implies that tumors are being diagnosed later than should be, which might be related to insufficient public awareness on the importance of periodic clinical and self-examination of the breasts. More efforts should be made to improve the efficacy and reachability of national screening programs, to ensure early diagnosis and most favorable patient outcomes.

Infiltrating ductal carcinoma is the most common type of BC, despite the difference in exact rates reported in different studies. Our study has found that 93% of HER2-positive patients had infiltrating ductal carcinoma, a rate that is higher than that reported in the Saudi Arabian 2015 Cancer Incidence Report (79%) [4]. Albasri et al. [25] has reported an 85% rate of infiltrating ductal carcinoma in a study conducted in Al-Madinah region, while Rudat et al. [7] has reported a rate of 91% in a similar study.

Considering that this was a retrospective study relying on secondary data collection, the data obtained may be subject to selection bias, since many patients could have been excluded due to the unavailability of HER2 testing results. In addition, the retrospective design might have affected the completeness of the data collected, due to the potential for missing data. Efforts to minimize bias and maximize data collection were done through selecting centers known to have high quality of clinical records-keeping. The comparison between HER2-positive and negative BC patients’ characteristics was not possible due to the lack of a control arm in this study.

This study is of primary importance since it included patients from 3 different centers distributed all over Saudi Arabia, which allows for a more representative assessment of the actual frequency of HER2 overexpression in the general BC patients pool, considering that most previous studies on BC epidemiology were done in limited regions in Saudi Arabia. The large sample size of this study and the long follow-up duration further enhanced the study’s representativeness. Our study explored the size of the burden of HER2 over-expressed BC diagnosed by traditional IHC and FISH and identified as a specific subgroup of patients that need to be managed accordingly. More genetically identified subgroups are likely to be identified in the future when modern techniques such as whole exome and RNA sequencing become established in routine clinical practice [30].

## Conclusions

This study has provided updated figures regarding HER2 overexpression in BC patients in Saudi Arabia: HER2 overexpression rate (29.9%) was within the range reported in previous studies conducted in Saudi Arabia. In addition, patients’ demographic and clinical characteristics in this study were also similar to those reported earlier, with a median age at diagnosis of 46 years and one third of patients being diagnosed with locally advanced/metastatic disease. This study has also highlighted prescription trends and survival outcomes in a sample of unique patients in Saudi Arabia.

## Data Availability

The data that support the findings of this study are available from [Clinserv CRO] but restrictions apply to the availability of these data, which were used under license for the current study, and so are not publicly available. Data are however available from the authors upon reasonable request and with permission of [Clinserv CRO].

## Abbreviations

BC: Breast cancer
ER: Estrogen receptor
PR: Progesterone receptor
HER2: Human epidermal growth factor receptor 2
ASCO/CAP: American Society of Clinical Oncology/College of American Pathologists
OS: Overall survival
IHC: ImmunoHistoChemistry
ISH: In situ hybridization
FISH: Fluorescent in situ hybridization
AEs: Adverse events
BMI: Body Mass Index
PFS: Progression-free survival
SOC: System organ classification
IRB: Institutional Review Board
